# Benefits and barriers associated with using cognitive–behavioral therapy to treat obsessive–compulsive disorder: a narrative review

**DOI:** 10.3389/fpsyt.2025.1593384

**Published:** 2025-07-28

**Authors:** Keiichiro Mukai, Yamanishi Kyosuke, Shun Ogino, Yukihiko Hosoi, Kazuhisa Hayashida, Hisato Matsunaga

**Affiliations:** Department of Neuropsychiatry, Hyogo Medical University, Nishinomiya, Japan

**Keywords:** cognitive-behavioral therapy, obsessive-compulsive disorder, narrative review, mental health apps, digital healthcare technology, internet interventions

## Abstract

There is a growing need for widely available, cost-effective, and low-intensity treatments for OCD. Although cognitive–behavioral therapy (CBT) is often the first line of treatment, barriers to providing CBT in OCD patients remain unresolved. In this narrative review, we summarize the current literature on the benefits and challenges of using CBT to treat OCD, review the potential of low-intensity, technology-based CBT programs, and identify issues related to the use of these new approaches. We identified articles to include in this narrative review by entering the following search terms into PubMed, PsychInfo, Web of Science, and Google Scholar: obsessive–compulsive disorder, OCD, cognitive–behavioral therapy, CBT, technolog*, digital. The final literature search was conducted on 13 July 2024, and after checking 68 potentially relevant studies according to our inclusion and exclusion criteria, we included 24 studies (14 review articles and 10 original articles) in the present review. We identified several main factors associated with the accessibility and effectiveness of CBT. Incentives for healthcare practitioners who undergo CBT training may increase the availability of this treatment option. Furthermore, treatment efficacy is related to patient treatment adherence, which may be enhanced by offering low-intensity and convenient treatment options such as digital CBT programs. These findings highlight both the potential and the current limitations of low-intensity and digital CBT approaches for OCD treatment. Although low-intensity and technology-based CBT programs can serve as relatively convenient, effective, and accessible treatment options, further research is needed to examine patient perceptions, and determine the most important characteristics of such programs for optimal treatment efficacy.

## 1 Introduction

### 1.1 OCD characteristics and prevalence

Obsessive–compulsive disorder (OCD) is a psychological condition in which individuals experience obsessive thoughts or urges that are unwanted or intrusive, or engage in compulsive or avoidant behaviors. Obsessions and compulsions are often distressing, disruptive to daily life, and difficult to ignore (1). Compared with the general population, individuals with untreated OCD have an increased mortality rate associated with both natural and unnatural causes, necessitating timely and effective disease management in this population (2). Because OCD onset often occurs in adolescence or early adulthood (1), the disorder is causally associated with a loss of productivity (3).

OCD is a relatively common chronic disorder, with a lifetime prevalence of 2% to 3% (4). Because it is often underdiagnosed, OCD is undertreated in the general population (4, 5). Unfortunately, individuals who develop OCD and remain untreated for prolonged periods of time tend to exhibit more severe symptoms, along with poorer prognosis. In addition, avoidance behaviors can make it difficult for affected individuals to leave their homes or interact socially, and this can lead to reluctance to seek treatment. Thus, the factors influencing treatment responsiveness overlap. Given the above-mentioned factors, early consultation and early initiation of treatment after onset are preferable for individuals with OCD. However, the accessibility of pharmacotherapy and cognitive–behavioral therapy (CBT), which are the first choices of treatment for OCD, can be limited (6, 7).

OCD is often comorbid with other psychiatric disorders, such as depression and anxiety, bipolar disorder, and attention deficit hyperactivity disorder (1, 8, 9). In particular, depression and social anxiety disorder are considered secondary comorbid symptoms following the onset of OCD. The implementation of standard treatment strategies for OCD could help to prevent the development of these comorbid symptoms.

### 1.2 Current use of CBT for OCD

Treatments for OCD include both psychological and pharmaceutical approaches. Most guidelines, including those of the American Psychiatric Association (10), recommend CBT, with exposure and response prevention and/or pharmacotherapy, as first-line treatments for OCD (11). Several recent reviews and meta-analyses have examined the effects of CBT on OCD symptoms (12, 13). Although there is strong evidence that CBT reduces OCD symptoms (11) and may increase remission rates (14), the quality and methodology of studies on the use of CBT for OCD has varied, necessitating further research with more standardized methods (15).

CBT is considered the most valuable treatment option for OCD because it is cost-effective compared with other treatment options. Most patients also prefer it to pharmacological therapy because of the potential side effects associated with the latter choice (3, 16). In addition, it can be provided online or via mobile applications, making it a convenient treatment option (17–20). Furthermore, while prescriptions for medication for conditions such as OCD can only be dispensed by psychiatrists in many countries, clinical psychologists and other healthcare practitioners who are unable to prescribe medication are often able to administer CBT. Thus, CBT may be an optimal treatment option for many patients compared with pharmaceutical alternatives. However, there are various challenges in implementing CBT, and a range of strategies have been used to improve CBT introduction and provision, including the use of low-intensity, technology-based approaches. There is currently a need for a narrative review of evidence regarding the benefits of and barriers to CBT for OCD and ways of promoting this treatment modality.

### 1.3 Aims of the review

In this narrative review, we first describe the importance of timely management of OCD, summarizing various treatment options for those with OCD, and new modalities such as CBT. We then summarize the current literature on the benefits and challenges of using CBT to treat OCD. Moreover, we describe the potential of low-intensity, technology-based CBT programs, providing evidence from literature on its benefits as well as identifying issues that need to be addressed in promoting the use of such approaches. Our aim in this narrative review was to provide a broad conceptual summary of the current status, trends, and evidence gaps in the use of CBT for patients with OCD.

## 2 Methods

The content of this review article is based on a narrative literature review conducted using online databases; PubMed, PsychInfo, Web of Science, and Google Scholar. In conducting this narrative review, we adhered to the SANRA (Scale for the Assessment of Narrative Review Articles) criteria to ensure methodological rigor and transparency (21). In our literature review, we focused on meta-analyses and original studies on the use of CBT and specifically technology-based CBT in the treatment of OCD. Key search terms included; obsessive–compulsive disorder, OCD, cognitive–behavioral therapy, CBT, technolog*, digital. Searches were performed using both US and UK spelling, and also with and without the terms “review” and “meta-analysis”. All key search terms were combined, using Boolean logic, such that one term related to OCD, one term related to CBT, and one term related to technology-based CBT, was searched, and subsequently, narratively reviewed. The final literature search was conducted on 13 July 2024.

**Figure 1:**
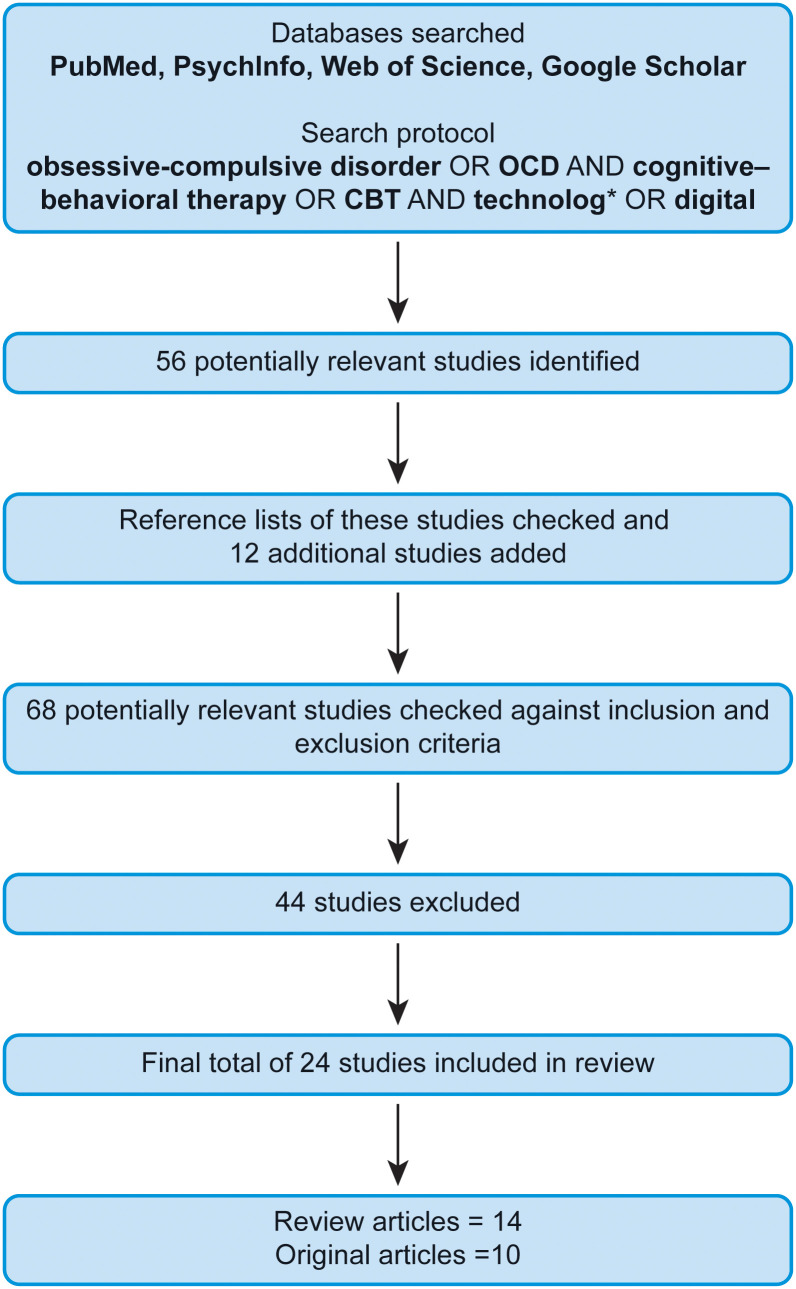
Flowchart of study selection. CBT, cognitive–behavioral therapy; OCD, obsessive–compulsive disorder.

The search initially retrieved 56 potentially relevant studies. We also assessed the reference lists of these articles to identify other essential studies (12 additional potentially relevant studies). We then checked those 68 studies against the inclusion and exclusion criteria, as follows. We applied basic inclusion parameters and focused on review articles and meta-analyses, but also considered original studies, on the use of different types of CBT for treating OCD for adults, to ensure relevance. We only examined studies conducted within the last 10 years, and studies of children were excluded. Following the search and exclusion/inclusion processes, 24 studies (14 review articles and 10 original articles) were included in the present narrative review. An overview of the study selection procedure is provided in **Figure 1**.

## 3 Barriers to the use of CBT for treating OCD

We assessed the included articles with the goal of identifying the main barriers and challenges reported in the use of CBT for treating OCD. We focused on the factors related to treatment participation and effectiveness, along with factors that have limited the widespread use of CBT in this patient population. **Table 1** summarizes the main characteristics of the 24 studies included in the review.

**Table 1:** Characteristics of the studies included in the review.

Original articles			
References	Study population	Design and measures	Main findings
Lundström et al, 2022 (66)	120 adults with OCD	RCT comparing therapist-guided iCBT, unguided iCBT, and individual face-to-face CBT	Therapist-guided iCBT: Inconclusive results compared with face-to-face CBT. Unguided iCBT: Statistically significantly inferior to face-to-face CBT, but noninferiority margin inconclusive.
Knopp-Hoffer et al., 2016 (39)	36 adults with OCD	Qualitative interviews on user perspectives of two low-intensity CBT interventions	Intervention uptake was good, but user individual differences affected patient satisfaction and perceptions of treatment effectiveness
Hwang et al., 2021 (47)	27 adults with OCD	Comparison between 6-week digital CBT intervention and traditional offline CBT	No difference in OCD symptoms between the conditions, but the digital CBT patients showed more symptom improvement
Wootton et al., 2024 (67)	216 adults with OCD	Open trial of the effectiveness of an 8-week self-guided iCBT intervention for OCD	Approximately one-quarter to one-third of participants showed clinically significant improvement at post-treatment and 3-month follow-up
Boisseau et al., 2017 (49)	21 adults with mild to moderate OCD	Open-pilot trial of the feasibility, acceptability, and preliminary efficacy of a mobile CBT app CBT for OCD	Post-treatment improvement in OCD symptoms
Gershkovich et al., 2021 (50)	33 adults with OCD	Pilot trial of the feasibility, acceptability, and clinical effects of an 8-week CBT program comprising in-person sessions and mobile app use	Participants showed post-treatment improvement in OCD symptoms and found the intervention feasible and acceptable
Tjelle et al., 2021 (30)	42 adults with OCD	Part of an RCT. 4-day exposure and response prevention intervention	Patients showed high adherence and reported fewer symptoms, better functioning, and greater well-being at follow-up
Gragnani et al., 2022 (31)	40 adults with OCD	Naturalistic outcomes study. Treatment procedure that included standard therapy, CBT, and psychotherapy	Reduction in OCD symptom interference, severity, and impairment after 9 months
Veale et al., 2016 (38)	472 patients with severe OCD attending a residential treatment facility	Open case series. Comparison of outcomes of a standard and intensive treatment program that included CBT	All residents showed significant improvement in OCD symptoms after treatment. There was no significant difference in outcomes between the two programs
Patel et al., 2018 (37)	40 adults with a principal diagnosis of OCD	Open trial of the acceptability, feasibility, and effectiveness of a 12-week internet-based CBT program	Significant reduction in OCD severity scores post-treatment and at 4-month follow-up. Participants also showed significant improvements in quality of life
Review articles			
References	Type and topic of review	Review focus and analysis	Main findings
Ferreri et al., 2019 (60)	Review of new technologies in OCD	Analysis of 62 articles on prediction, assessment, and interventions in new technologies for OCD	Although the role of some new technologies remains to be defined, smartphone apps and web screening tools compared favorably with clinical interviews in detecting OCD symptoms. Technology-supported CBT is not necessarily more effective, and CBT supported by smartphone, internet, or computer may not be more effective than practitioner CBT, but it is cost effective and patients find it easy to use
Stefanopoulou et al., 2019 (62)	Review of digital interventions for anxiety disorders	Analysis of 68 RCTs to compare the effectiveness of digitally delivered psychological therapies with control conditions and/or other psychological interventions for anxiety disorders (including OCD)	Some studies indicated significant within-group improvement in OCD, but not necessarily between-group improvement. Lack of strong evidence because of methodological differences across trials
Waks et al., 2024 (59)	Systematic review and meta-analysis of the acceptability of iCBT for adults with OCD	Meta-analysis of 17 quantitative studies using a clinician-guided or self-guided iCBT intervention for adults with OCD	Self-guided iCBT were reported as being less acceptable than clinician-guided interventions. However, the authors consider the findings to be preliminary because of the low power of the analysis
Hoppen et al., 2021 (55)	Systematic review and meta-analysis of low-intensity technology-delivered CBT for OCD	Meta-analysis of 18 RCTs using computer- or mobile phone-based iCBT interventions for adults with OCD	Compared with participants in passive control groups, those receiving iCBT interventions reported improvements in OCD symptoms
Fordham et al., 2021 (12)	Meta-review of systematic reviews and panoramic meta-analysis of RCTs of CBT interventions for a range of conditions (including OCD)	Analysis of 494 reviews (378 of which were on adults) of the effect of CBT on health-related quality of life	Most reviews were of low quality and focused on face-to-face CBT. Few were from Asia, South America, or Africa. A modest benefit across conditions was found for CBT
Henrich et al., 2023 (26)	Systematic review of training in CBT	Analysis of 51 publications on the effectiveness of research in CBT training, including specific training elements and costs	Instructor-led training and self-guided web-based training moderately improve competence. However, training-related gains in competence are influenced by therapists’ levels of previous training and experience
Frank et al., 2023 (19)	Scoping review of literature on wearable and mobile technologies for evaluating and treating OCD	Analysis of 25 articles on the use of wearable devices and smartphone-based devices or apps in OCD assessment, monitoring, and treatment using active, passive, or mixed data collection	The reviewed studies used a range of mobile or digital interventions and the findings identified an increase in use of wearable sensors and mobile apps in evaluating and treating OCD over the last 15 years. There is evidence for a general improvement in OCD symptom burden following digital/mobile treatments
Leeuwerik et al., 2019 (32)	Systematic review and meta-analysis of the challenge of patient adherence to CBT for OCD	Analysis of 123 studies to identify the magnitude, moderators and reasons for poor patient adherence to CBT for OCD	Most studies reported moderate to good adherence to between-session CBT tasks (pooled rate of 15.6% of patients refused CBT and 15.9% dropped out of treatment). CBT was significantly associated with OCD symptom reduction
Strouphauer et al., 2023 (44)	Systematic review of the cost-effectiveness of interventions (including CBT) for OCD	Analysis of 18 cost-effectiveness studies and narrative synthesis of the findings	The findings of studies of CBT showed that iCBT demonstrated clinical superiority and cost-effectiveness compared with treatment as usual. However, compared with in-person CBT, the cost-effectiveness of iCBT was unclear and efficacy was likely lower
Hiranandani et al., 2023 (54)	Brief review of evidence-based interventions for obsessive–compulsive and related disorders and related disorders	Literature review of current evidence-based digital interventions (focusing on internet- and app-based interventions) for OCD and identification of areas for future research	There is evidence that digital CBT interventions (including internet- and app-based programs) are as effective as face-to-face treatments. Such interventions can address barriers to treatment and are considered flexible, accessible, and cost-effective. However, more evidence is needed regarding efficacy, patient engagement, and data privacy
Kumar et al., 2017 (56)	Review of the of the effectiveness of iCBT for psychiatric disorders (including OCD)	Analysis of 373 articles on internet-based, web-based, and mobile-based CBT, focusing on effectiveness, cost-effectiveness, and impact in rural and urban settings	The findings showed that iCBT is effective and cost-effective in treating mental health disorders, and is applicable across various settings and populations
Elsouri et al., 2024 (2)	Review of recent OCD management and treatment strategies (including CBT)	Comparison of the benefits and limitations of the main treatments for OCD; namely, psychotherapy (including CBT), pharmacotherapy, and neurological approaches	The authors conclude that CBT has shown effectiveness and clinical benefits for patients with OCD. However, they note that barriers to this treatment modality include patient burdens of time and cost
Cooper et al., 2022 (70)	Scoping review of clinical interventions for anxiety and OCD that have integrated technology	Analysis of data from 117 primary studies, including examination of quantity, type, and purpose of technological innovations used and outcomes such as symptom changes	All studies excerpt one reported a significant change in symptoms, but few demonstrated significant differences in the intervention compared with the control group. Few studies reported implementation factors
Öst et al., 2022 (13)	Systematic review and meta-analysis of CBT for OCD in adults treated in routine clinical care	Analysis of data from 29 studies (including 8 RCTs) in terms of CBT effectiveness (as compared with efficacy), study quality, and treatment outcome moderators	Large within-group effects were found for CBT, and the effectiveness and efficacy were similar. However, most studies showed substantial risk of bias. Nevertheless, the authors conclude that CBT is an effective treatment for OCD in routine clinical care

CBT, cognitive–behavioral therapy; iCBT, internet-based CBT; OCD, obsessive–compulsive disorder; RCT, randomized controlled trial.

In terms of the likelihood that patients will seek treatment for OCD, some studies have highlighted the importance of the role of negative stigmatization associated with obtaining a diagnosis of OCD (22, 23), and described a lack of confidence among patients regarding the efficacy of psychiatric treatment in reducing OCD symptoms (6). The studies identified a number of factors that may affect the provision of CBT for OCD patients. For instance, one discussed the prioritization of pharmacotherapy in healthcare and the lack of availability of trained therapists who could deliver CBT (3). A recent literature review of different treatments for OCD showed that although CBT may be more efficacious in reducing the severity of OCD symptoms, pharmacotherapy may be more cost-effective and associated with greater patient adherence (2).

Various approaches developed to increase the number of trained therapists and subsidize the cost of treatment for patients appear to have increased the number of patients seeking CBT. For example, the implementation of the Improving Access to Psychological Therapies (IAPT) program in the UK has provided training for therapists and subsidized the cost of treatment (24–26). This indicates that there is a high demand for CBT and thus an unmet need for treatment for this patient population.

Some psychiatrists may not be familiar with CBT or with the many different types of CBT that have been developed. Additionally, therapists who complete CBT training programs may need to acquire a substantial amount of practical experience before they can offer sufficient quality and effective CBT (27). Accordingly, although they may be challenging to devise, effective strategies are needed to encourage prospective therapists to undergo competency-based training in the implementation of CBT. There is some evidence to suggest that incentives offered to therapists may increase their implementation of CBT. For example, Beidas et al. (28) found that both social incentives and financial rewards were effective in incentivizing community mental health therapists to use CBT. Although there is a dearth of research on this topic, recent studies suggest that the use of more active training strategies (e.g., role play) and online training could help to increase practitioners’ knowledge, and subsequently increase implementation, of CBT (29, 30). Additionally, governmental policies that prioritize training in CBT methods for medical practitioners may facilitate increased access for patients.

The studies included described the following main factors associated with the effectiveness of CBT. First, patient adherence was associated with the effectiveness of CBT for treating OCD (31, 32). However, more research is needed to determine which aspects of patient adherence are most important for treatment success (33). Second, an important factor that affected patient adherence to conventional face-to-face CBT programs was the patient’s ability and willingness to travel to attend a weekly course that may have as many as 10–15 sessions. Geographical and financial constraints can make travel burdensome for patients, particularly those who also have work and family commitments, or who experience avoidance behaviors (34). Therefore, there is a need to develop a greater range of CBT programs that offer different delivery modes and levels of intensity. Finally, some forms of CBT, such as exposure and response prevention, can be challenging for patients because they require them to face the object of their fear or anxiety (32).

## 4 Strategies for increasing CBT provision and adherence

**Figure 2:**
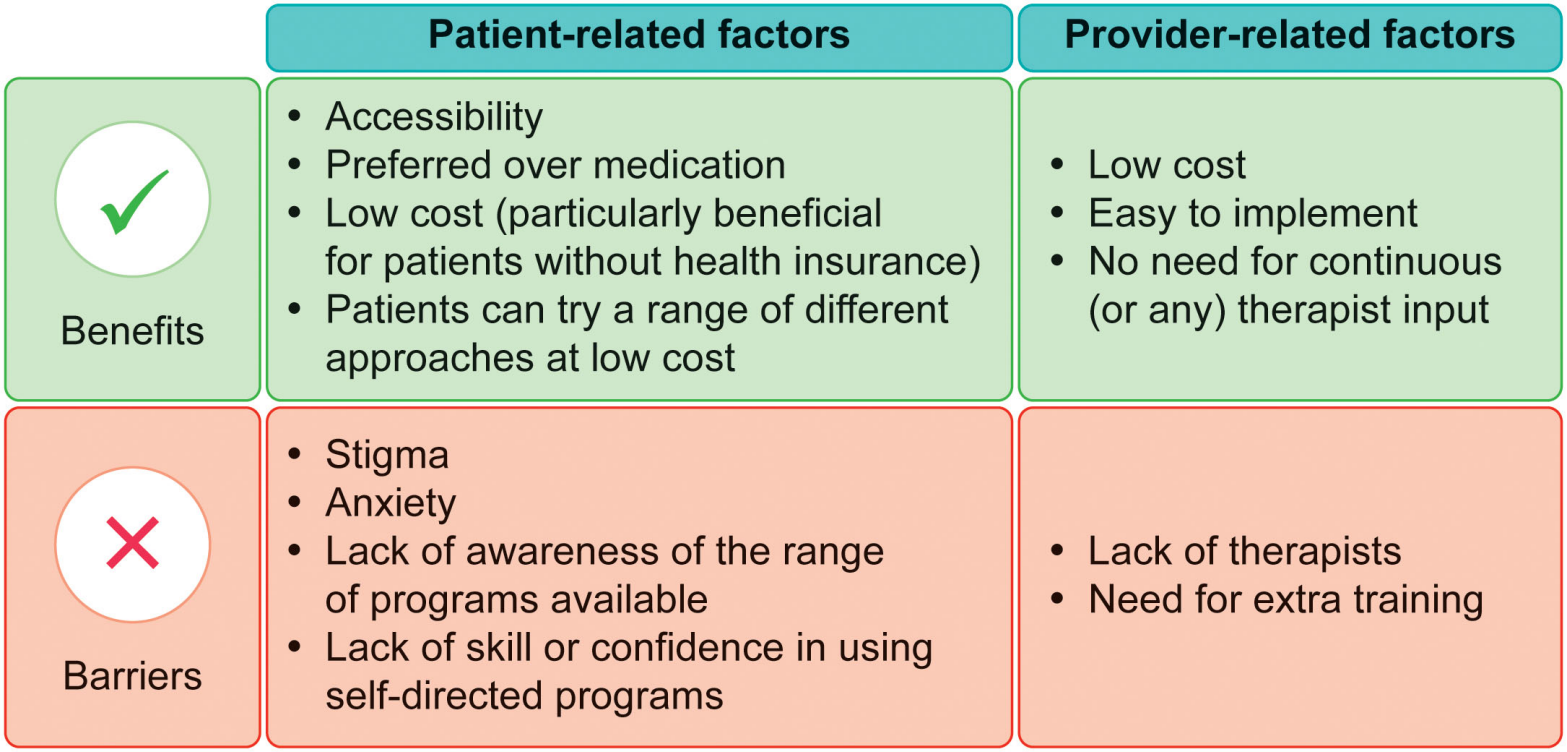
Benefits of, and barriers to, the provision of low-intensity cognitive–behavioral therapy for obsessive–compulsive disorder.

Several of the studies identified factors that could increase the provision of, and adherence to, CBT for people with OCD (**Figure 2**). We focused particularly on the use of low-intensity and technology-based CBT approaches. **Table 2** provides examples and summarizes the characteristics and findings of several studies (including quantitative and qualitative studies) that focused particularly on these types of approaches.

**Table 2:** Examples of studies on low-intensity, technology-based cognitive–behavioral therapies (CBT) for obsessive–compulsive disorder (OCD).

Therapy	Study	Target group	Results
Guided and unguided internet-based CBT	Randomized controlled trial by Lundström et al., 2022 (66)	Adults with OCD in Sweden	Therapist-guided iCBT: Inconclusive results compared with face-to-face CBT. Unguided iCBT: Statistically significantly inferior to face-to-face CBT, but noninferiority margin inconclusive.
Guided self-help and computerized CBT	Qualitative study by Knopp-Hoffer et al., 2016 (39)	Adults with OCD in the UK	Individual differences between users greatly affected patient satisfaction and perceptions of treatment effectiveness: patients who valued therapist support were not happy with computerized CBT.
Internet-delivered CBT and clinician‐guided interventions	Meta-analysis of 17 quantitative studies by Waks et al., 2024 (59)	Adults with OCD	Acceptability moderator analyses indicated lower levels of acceptability for self‐guided internet-delivered CBT interventions versus clinician‐guided interventions. However, these results should be considered preliminary.
A mobile app CBT program and traditional CBT	Pilot comparative study by Hwang et al., 2021 (47)	Adults with OCD in Korea	No change in OCD symptoms, but those who completed the digital CBT program had lower levels of anxiety compared with the offline CBT group. Both treatment groups showed improved functional connectivity.
Technology-delivered CBT and passive control groups	Meta-analysis of 18 randomized controlled trials by Hoppen et al., 2021 (55)	Adults with OCD	Lower scores on various scales of OCD symptoms in the treatment versus control groups indicating treatment efficacy.
CBT video-conferencing treatment	Feasibility study by Hollmann et al., 2021 (48)	Children with OCD	Post-treatment decrease in OCD symptoms, high patient and parent rating of approval for app system.
A mobile app CBT program	Single-arm intervention study by Boisseau et al., 2017 (49)	Adults with mild to moderate OCD in the USA or Canada	Post-treatment improvement in OCD symptoms and anxiety.
CBT program involving person sessions followed by independent use of a mobile app	Open trial by Gershkovich et al., 2021 (50)	Adults with OCD in the USA	Post-treatment improvement in OCD symptoms.

### 4.1 Low-intensity CBT approaches

New low-intensity CBT approaches have been developed and studied since the late 2000s (35). They offer treatment characterized by low clinical contact or contact with non-specialists, and/or the use of technology-based materials (36). Such programs appear to correspond with the UK NICE Guidelines for OCD, which recommend the use of low-intensity CBT for patients with milder forms of OCD (37). However, these programs do not appear to be widespread, meaning that targeted efforts are needed to increase the availability and attractiveness of low-intensity CBT approaches for people with OCD (38).

The articles examined indicated that the use of low-intensity CBT approaches could help to address cost- and geographic-related constraints experienced by some patients by providing alternatives to conventional CBT formats. Although residential or inpatient high-intensive CBT programs may be more effective for patients with severe OCD symptoms (39), low-intensity CBT is well suited to patients with moderate to mild OCD (40). Because of the lower frequency of treatment, low-intensity approaches can lessen the burden on patients to attend sessions, thus increasing the accessibility and sustainability of CBT for a more diverse range of patients.

Low-intensity CBT programs may also be advantageous compared with alternative treatment options within the context of human resource costs. Conventional face-to-face CBT programs delivered by specialists typically involve 10–15 weekly sessions that are approximately 30–60 minutes in duration (1). Accordingly, the high human resource cost involved in this type of delivery impedes the nationwide implementation of easily accessible standard CBT programs (41). Low-intensity CBT, particularly technology-based programs, can be delivered by qualified healthcare workers or support workers instead of mental health professionals. Furthermore, these programs can be shorter and more accessible than traditional CBT. For instance, one study found that low-intensity CBT programs were typically completed in approximately half the time of conventional programs (36). Indeed, the provision of CBT could be increased if more cost-effective methods were used, such as with the MindSpot program in Australia (42). However, a sound rationale for such methods is needed. While several treatment modalities are available for treating OCD, these must be developed according to the specific context of the medical system, clinical settings, and geographical factors. Some emerging evidence has begun to address this. In Japan, for instance, Matsumoto et al. (2022, 2024) reported favorable clinical and economic outcomes for guided internet-based CBT in OCD (20, 43). While these interventions were not delivered within Japan’s formal insurance-based medical system—given that psychologists are not yet fully reimbursed as providers—they still offer meaningful regional data. Several studies have reported on the cost-effectiveness of CBT interventions, including low-intensity and digital formats (44, 45). However, few studies have directly compared the cost-effectiveness of different CBT delivery methods, such as face-to-face versus internet-based programs (46). Therefore, more research is needed regarding the cost-effectiveness of low-intensity CBT in diverse healthcare settings.

### 4.2 The potential of technology-based approaches in delivering low-intensity CBT

Internet-based and mobile app CBT programs generally include text, audio, and video components, and often have homework, along with feedback for completed homework assignments (47, 48). A number of these types of CBT programs have been reviewed by researchers (49–52). Additionally, the International OCD Foundation has reviewed several apps for the treatment of OCD (53). The clinical effectiveness of technology-based CBT approaches has also been demonstrated in several randomized controlled trials. For example, Wootton et al. (2013) demonstrated that therapist-guided internet-based CBT (iCBT) led to large reductions in OCD symptoms compared with a waitlist control (44). Building on these findings, Matsumoto et al. (2022) conducted a cost-effectiveness analysis of guided internet-based CBT in Japan, demonstrating both clinical efficacy and economic efficiency (20). Furthermore, a 24-month follow-up study by the same group confirmed the long-term effectiveness and cost-effectiveness of this intervention, underscoring its sustained clinical utility and viability (43). Herbst et al. (2014) found that an internet-based writing intervention was beneficial in reducing the severity of OCD symptoms (54). More recently, Wu et al. (2023) showed that iCBT was non-inferior to group CBT in terms of both symptom improvement and treatment adherence (45). In parallel with these research-based interventions, a growing number of publicly available CBT apps have emerged (55). Most of these apps are free or very low cost, are self-directed, and use exercises and strategies to train users to change maladaptive thoughts and behaviors. Accordingly, the number of programs is growing rapidly, and although some have been reviewed (and are evidence-based), others have yet to be reviewed. CBT programs delivered via mobile apps are generally lower in intensity than iCBT, with minimal reliance on human resources. Mobile app CBT programs also have fewer time restrictions, as treatment sessions do not have to be scheduled. Additionally, features unique to mobile phones, such as reminder functions, may be useful for supporting adherence. These advantages should be considered in future research.

Recent reviews of digital mental health apps and interventions have found that, although digital and mobile treatments vary, they generally lead to a decrease in symptom burden (19). Furthermore, they may address existing gaps in healthcare by offering scalable, low-stigma, and cost-effective solutions (56). Although there is evidence for the effectiveness of these approaches (56–58), user preferences can affect patient satisfaction and engagement (40). Furthermore, the effectiveness of some technology-based applications has not been validated (59). Another challenge with the use of technology-based CBT approaches is ensuring equality of access to technological resources. Some patients may not have easy access to technology, and older patients in particular may find it more difficult to access digital interventions (60). Furthermore, collaboration between developers and healthcare professionals has been insufficient to lead to optimal design choices for technology-based CBT programs (59).

Despite the above-mentioned challenges, there are a number of additional benefits to using technology-based CBT programs. For instance, technology-based CBT programs seem to be well accepted by OCD patients (61) and may be especially attractive to patients who are reluctant to engage in face-to-face therapy (57) or who live a long distance from a therapist. Given the challenges associated with effecting the widespread adoption of in-person CBT programs, digital approaches are likely to offer feasible alternatives. Therefore, there is a need for research regarding the equivalency of interpersonal CBT and digital treatments, as well as approaches for mitigating potential differences. Compared with face-to-face interventions, digital treatment strategies are easier to implement and are well accepted by patients (62) and healthcare personnel (63). Given the advantages of iCBT in terms of cost and accessibility (46, 58), the value of such digital approaches should not be overlooked. Challenges to the assessment of digital approaches to treating OCD and other disorders include determining the influence of a range of factors (such as therapist involvement level and patient characteristics) on the effectiveness of these methods (64). Therefore, better assessment techniques for determining the effectiveness of digital approaches to OCD are needed. These include determining the influence of various factors, such as therapist involvement level and patient characteristics, on treatment outcome (64).

The relatively recent upsurge in low-intensity digital treatments for psychiatric conditions has highlighted the need for common standards and consensus regarding evaluations of the effectiveness of such treatments (65). Several factors (e.g., the influence of the therapist–patient relationship and a lack of clarity regarding the mechanisms underlying the treatment effect) make it difficult to design control conditions for psychological interventions (including digital interventions) such as CBT (65). Despite these factors, the US Food and Drug Administration recommends the use of randomized controlled trials with sham devices to assess the effectiveness of digital treatments (65, 66). However, the definition of a sham device is ambiguous (65, 67), and the design and validation of appropriate sham devices that are not detectable by patients remains a challenge. Accordingly, it is difficult to blind participants in trials of digital CBT devices to their trial condition, thus limiting the unbiased assessment of the treatment effect. To address this, new applications should be tailored to address the unmet medical needs of patients with OCD, and assessed by comparing them with programs that use different therapeutic styles implemented in various medical settings.

OCD is a heterogeneous psychiatric condition (1) with symptoms that vary according to severity and other factors such as comorbidity with other disorders. Therefore, when developing novel CBT approaches, it is important to understand the unique characteristics of specific patient populations. This would facilitate the tailoring of interventions to specific subsets of patients (40), and make it easier to generate clear protocols for the assessment and validation of the clinical utility of different low-intensity CBT programs. The examined studies indicate that substantial challenges remain in implementing digital CBT programs, such as providing incentives to encourage therapists to engage in further training and determining user competence to complete self-guided programs (40).

## 5 Future directions

Further research is needed to address the gaps in the literature regarding the use of low-intensity, technology-based CBT approaches for treating OCD. For instance, research is needed regarding the potential differences in the costs and efficacy of self-guided and guided digital therapies for OCD (68). There is a gap in the literature with respect to the specific predictors of the outcomes of digital OCD therapies (69). Additionally, research on patient preferences for self-guided vs. guided treatment is lacking (69, 70). Addressing this issue could help researchers design treatment programs that are more attractive to patients. In particular, there is a need for more qualitative studies on various aspects of user perspectives of technology-based CBT approaches (40, 71), including overall user satisfaction.

Individual differences may play an important role in determining the selection and effectiveness of low-intensity digital approaches. Evidence suggests that users vary in terms of the value they place on aspects such as professional support in self-guided programs (40, 72). Accordingly, more research is needed to understand the requirements and expectations regarding therapy in OCD patients, along with the desirability of tailored approaches. Finally, there is a dearth of research regarding the appropriate use of sham devices in randomized controlled trials of technology-based CBT. Accordingly, further investigations are needed to determine the optimal ways to use sham devices in treatments for psychiatric problems, and to determine the effects of sham controls on psychiatric outcomes (67).

Finally, low-intensity CBT is well suited for initial treatments and in mild cases of mental health disorders, providing an accessible form of therapy that can effectively manage symptoms without intensive intervention. However, traditional CBT, which involves more frequent and intensive sessions, may be more appropriate for moderate cases, patients with comorbid psychiatric conditions, or those receiving concurrent pharmacotherapy. This differentiation in therapeutic intensity highlights the importance of developing a range of therapeutic approaches, such as stepped care models, that match treatment modalities to patient groups based on severity and specific needs. To maximize the use of medical resources and provide tailored treatment pathways that enhance patient outcomes, it is essential to implement such frameworks in healthcare systems and develop appropriate guidelines.

## 6 Conclusions

The studies examined in this narrative review indicate that there is a growing demand for widely accessible, cost-effective, and low-intensity treatments for OCD. However, there are several diverse barriers to the provision of in-person CBT in OCD patients. Low-intensity, technology-based CBT programs hold promise as accessible and affordable treatment options. However, more research is needed, with a focus on the differences between guided and self-guided programs, patient perceptions of treatment options, strategies for controlled trials, and the influence of individual patient differences, to realize the potential of such approaches.
